# Effect of Imatinib on Bone Marrow Morphology and Angiogenesis in Chronic Myeloid Leukemia

**DOI:** 10.1155/2019/1835091

**Published:** 2019-01-01

**Authors:** Neetu Pandey, Geeta Yadav, Rashmi Kushwaha, Shailendra Prasad Verma, Uma Shankar Singh, Ashutosh Kumar, Prabhaker Mishra

**Affiliations:** ^1^Department of Pathology, King George's Medical University, Lucknow, U.P. 226003, India; ^2^Department of Clinical Hematology, King George's Medical University, Lucknow, U.P. 226003, India; ^3^Department of Biostatistics & Health Informatics, Sanjay Gandhi Post Graduate of Medical Sciences, Raebareli Road, Lucknow, U.P. 226014, India

## Abstract

**Background and Objectives:**

Chronic myeloid leukemia (CML) is characterized by hyperproliferation of myeloid precursors, increased fibrosis, and neoangiogenesis in the bone marrow. Imatinib inhibits BCR-ABL tyrosine kinase produced due to reciprocal translocation t(9;22) in neoplastic CML cells. It reduces hyperproliferation of myeloid precursors and has been found to affect bone marrow fibrosis and angiogenesis. This study was done to assess the effect of imatinib on bone marrow morphology and angiogenesis in CML.

**Methods:**

31 newly diagnosed CML patients were evaluated before and after 3 months of imatinib therapy. A marrow morphological response (MMR) score was used to assess marrow cytological and histological features including grade of fibrosis. Mean microvessel density (MVD) was also assessed. Hematological parameters and BCR-ABL transcript levels were assessed in the peripheral blood.

**Results:**

86.21% of patients showed decrease in marrow cellularity with normalization of M:E ratio. 72.42% of patients had decrease in grade of fibrosis and 17.24% showed no change while 10.34% of patients showed progression of fibrosis grade. Patients with MMR score ≥ 2 (n=4) and those with progression of fibrosis grade (n=3) showed suboptimal molecular response (BCR-ABL transcripts > 10%). Pretherapy mean MVD of patients (14.69 ± 5.28) was higher than that of controls (6.32 ± 1.64). A significant reduction of 66.51% was observed in posttherapy mean MVD (4.98 ± 2.77) of CML patients (p<0.001).

**Conclusion:**

Imatinib therapy in CML not only decreases marrow cellularity, but also helps towards normalization of bone marrow microenvironment by reducing fibrosis and angiogenesis.

## 1. Introduction

Chronic myeloid leukemia (CML) is a myeloproliferative neoplasm characterized by excessive accumulation of apparently normal myeloid cells in the bone marrow and peripheral blood. It is associated with the presence of Philadelphia chromosome, which is the result of a reciprocal translocation between chromosomes 9 and 22 resulting in the formation of BCR-ABL chimeric protein which has uncontrolled tyrosine kinase activity [1]. Besides increase in myeloid cells, the bone marrow microenvironment in CML is also altered. There is an increase in collagen type III (reticulin fibrosis) as well as an increase in angiogenesis [2]. Imatinib mesylate is a selective BCR-ABL protein tyrosine kinase inhibitor which acts by binding to the ATP binding site of BCR-ABL tyrosine kinase, causing its inactivation and thereby effecting apoptosis of leukemic cells which leads to improvement in bone marrow hematological and morphological parameters [3, 4]. Imatinib also inhibits other tyrosine kinases, namely, platelet derived growth factor receptor (PDGFR) and c-KIT [3]. Imatinib also has effects on the bone marrow stroma and microenvironment. A decrease in bone marrow fibrosis has been observed in CML patients on imatinib therapy [2]. Recent studies have emphasized on the antiangiogenic property of imatinib. Angiogenesis plays an important role in progression of solid tumors and angiogenesis induction has been described in several hematological malignancies including leukemias [5]. The present study was done with aim to assess morphological changes and changes in angiogenesis in the bone marrow in patients of CML on imatinib mesylate therapy.

## 2. Materials and Methods

This was a prospective study done over a period of one year between July 2016 and June 2017. The study was approved by the Institutional Ethics Committee. A total of 31 patients of CML were enrolled in this study. Written informed consent was taken from all the patients. Detailed history was obtained from each patient. Detailed clinical examination and blood and bone marrow examination were done in all patients. BCR-ABL status estimation was done by FISH or real-time PCR (RT-PCR). All patients showed presence of BCR-ABL translocation at the time of diagnosis.

All patients received a single daily dose of 400-600 mg of imatinib mesylate throughout the study period according to the European LeukemiaNet guidelines (2006) [6]. All the patients in chronic phase received a standard dose of imatinib (400 mg/day). Patients in accelerated and blast phase received 600 mg/day of imatinib. Hematological response, molecular response, and morphological response were evaluated after 3 months of therapy and assessed based on standard criteria.

1. **Hematological response** was graded as follows:
  - Complete hematological response (CHR): WBC count < 10 x 10^9^/L, platelet count < 450 x 10^9^/L, no immature cells in peripheral blood, and no splenomegaly.
  - Partial hematological response (PHR): ≥ 50% reduction of WBC count, presence of immature granulocytes in peripheral blood, and persistence of splenomegaly.
  - Nil hematological response (NHR): response worse than partial.
2. **Molecular response** was assessed by quantitative RT-PCR from peripheral blood samples and graded as per European LeukemiaNet Response Definitions and Monitoring Recommendations (2013) [7].
  - Complete: BCR-ABL-1 transcripts nonquantifiable and nondetectable.
  - Major: BCR-ABL-1 transcripts ≤ 0.10% on the international scale.
  - Early: BCR-ABL-1 transcripts ≤ 10% at 3 months.
3. **Morphological response** was assessed by cytological features in bone marrow aspirate smears and histological features in bone marrow core biopsies. Findings were evaluated by two independent pathologists.
  - Histologic features assessed were cellularity, myeloid to erythroid (M:E) ratio, fibrosis, and abnormal megakaryocytes.
  - Cytologic features assessed included nature of tap, percentage of blasts, and percentage of basophils.

A scoring system described by Lugli A et al. [8] was used for the grading of morphologic response as described in Table 1. Points for histological and cytological features were added.

For the assessment of fibrosis, Gomori's reticulin stain was performed on bone marrow biopsy sections to demonstrate myelofibrosis and was graded as per European consensus on grading bone marrow fibrosis and assessment of cellularity [9].

  Grade 0: scattered linear reticulin with no intersections.
  Grade 1: loose network of reticulin throughout the section.
  Grade 2: diffuse and dense increase in reticulin with extensive intersections with focal collagen bundles.
  Grade 3: diffuse and dense increase in reticulin with extensive intersection and coarse bundles of collagen associated with osteosclerosis.
(4) **Assessment of angiogenesis:** bone marrow biopsy sections (4 *μ*M thick) were subjected to immunohistochemical identification of microvessels by monoclonal mouse anti-human anti-CD34 antibody (clone QBEND10, Dako, Agilent). The antibody was used at a dilution of 1:400 for 90 minutes. This was followed by application of labeled streptavidin-biotin peroxidase method. Counting of microvessels was done by two observers independently in all bone marrow biopsies using a Leica light microscope. The bone marrow biopsy sections were screened at 100x magnification for areas of maximum numbers of CD34 positive endothelial cells (hotspots). Randomly selected hotspots were screened at 200x to clearly identify microvessels. Microvessel density (MVD) was measured in these fields at high power (400x) magnification and the mean MVD was calculated by taking the average of four hotspots per slide. All the hot spots were marked and seen by both observers and a consensus was reached whenever there was a discrepancy in MVD assessment. Only brown stained CD34 positive endothelial cells or clusters with or without a lumen were considered as countable microvessels. Care was taken to exclude CD34 positive vessels with muscular walls and CD34 positive scattered clusters of blasts and other CD34 positive artefacts such as skin tissue in BM.

## 3. Statistical Analysis

Statistical analysis was done using Statistical Package for Social Sciences, version 23 (SPSS-23, IBM, Chicago, USA). Normality of continuous data was assessed. Normally distributed continuous data were presented in Mean ± Standard Deviation (Mean ± SD), while categorical data were presented in frequency and percentage (%). Independent samples t-test was used to compare means between two groups while Fischer's exact test was used to compare proportions/test associations between two attributes. Paired samples t-test/McNemar's Chi-Square test was used to test the mean difference/difference in proportions between pre and post observations. p value < 0.05 was considered as statistically significant.

## 4. Results

In this study, 31 patients of CML (age range 15-65 years, male: female ratio = 1:1.07) were subjected to imatinib therapy. Of these 31 patients, 25 were in the chronic phase of CML, 2 in the accelerated phase, and 4 in the blast phase at the time of diagnosis. All 31 patients were symptomatic at the time of presentation and the most common symptom was easy fatigability (97%). 22 out of 31 patients (70.96%) patients had splenomegaly at the time of presentation with mean spleen size (cm) below costal margin = 6.59 ± 2.61. In 29.03% (n=9) of patients, spleen was not palpable at diagnosis. The mean haemoglobin concentration (gm/dL) was 9.66 ± 2.54 (range = 4.7-16.2, median =10.05). The mean total leucocyte count (lac/mm^3^) of patients was 1.36 ± 0.81 (range = 0.3 – 3.0, median =1.30). The mean basophil percentage of patients was 3.71 ± 3.79% (range = 0-18%, median = 2.50%). 3-month follow-up of 29 patients was obtained. 2 patients could not be followed up as they died within 3 months of onset of treatment.

### 4.1. Post Therapy Spleen Size

Spleen was not palpable in 21 out of 29 (72.41%) patients after therapy. 8 out of 29 patients (27.58%) had splenomegaly after 3 months of imatinib therapy with mean spleen size (cm) below costal margin = 4 ± 2.13 (range 2-8 cm).

### 4.2. Hematological Response

Out of 29 patients, 15 patients (51.7%) achieved CHR and 12 patients (41.3%) achieved PHR. 2 patients (7%) showed NHR and remained in chronic phase as initially. A summary of hematological response is shown in Table 2.

### 4.3. Molecular Response

BCR-ABL fusion transcripts were detected in all 31 patients at the time of diagnosis. At follow-up of 3 months, BCR-ABL fusion transcript values of only 26 patients could be obtained. 5 (19.24%) out of 26 patients achieved complete molecular response. 10/26 patients (38.46%) showed early molecular response. In 11/26 patients (42.30%), the BCR-ABL transcript levels were ≥ 10%.

### 4.4. Marrow Morphological Response (MMR)

**Figure 1:**
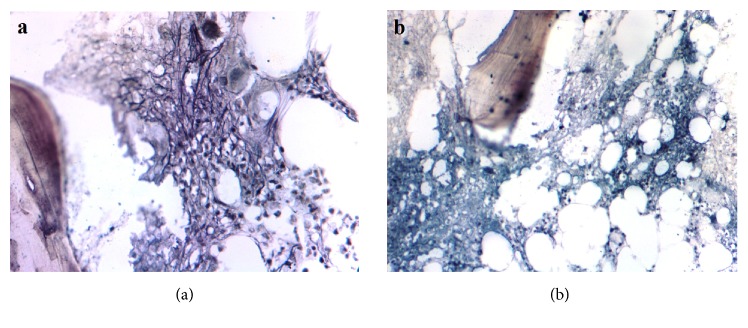
(a) Pre therapy histological section of bone marrow biopsy stained by Gomori reticulin stain (400x magnification) showing diffuse and dense increase in reticulin with extensive intersections with coarse bundles of collagen (grade 3 fibrosis). (b) Post therapy histological section of bone marrow biopsy of the same patient (400x magnification) showing scattered linear reticulin with no intersections (grade 0 fibrosis).

Majority of cases (86.21%) showed decrease in cellularity with normalization of M:E ratio. 82.76% cases show reduction in abnormal megakaryocytes in bone marrow. Basophil count >1% was seen in 70.96% at the time of diagnosis, which was seen in only 17.24% cases after therapy. Blast count >5% was seen in 32.25% of cases at the time of diagnosis, which was reduced to 3.44% cases after imatinib therapy. Pretherapy grade of fibrosis of majority of patients (61.29%) was Grade 2, followed by Grade 3 (25.81%) and Grade 1 (12.9%). At follow-up, Grade 1 was most common grade of fibrosis (41.38%) followed by Grade 2 (31.03%), Grade 0 (20.69%), and Grade 3 (6.90%). 21 (72.42%) patients showed decrease in grade of fibrosis at 3 months after imatinib therapy. 5 (17.24%) patients had no change in fibrosis grade. 3 (10.34%) patients showed progression of fibrosis after 3 months of imatinib therapy. Decrease in grade of fibrosis after 3 months of imatinib therapy was found to be statistically significant (p<0.001).  Figure 1 shows change in grade of fibrosis in a patient on imatinib therapy.

**Figure 2:**
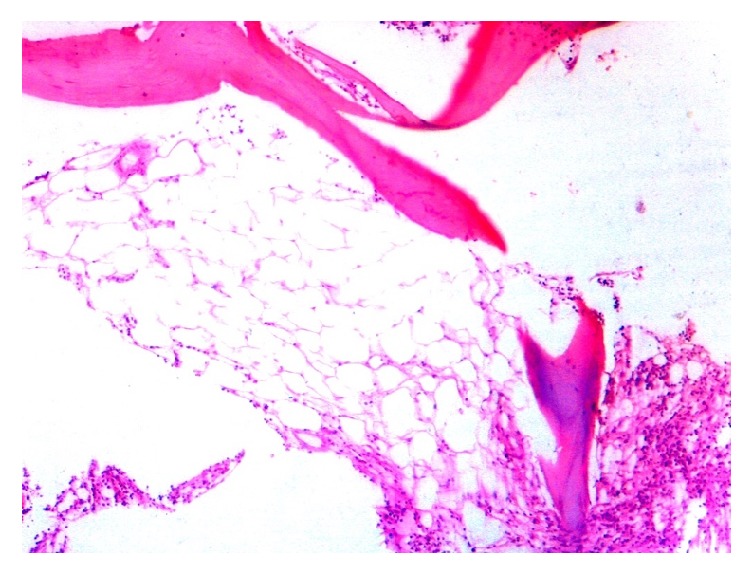
Post therapy histological section of bone marrow biopsy showing marked hypocellularity (400x magnification).

**Figure 3:**
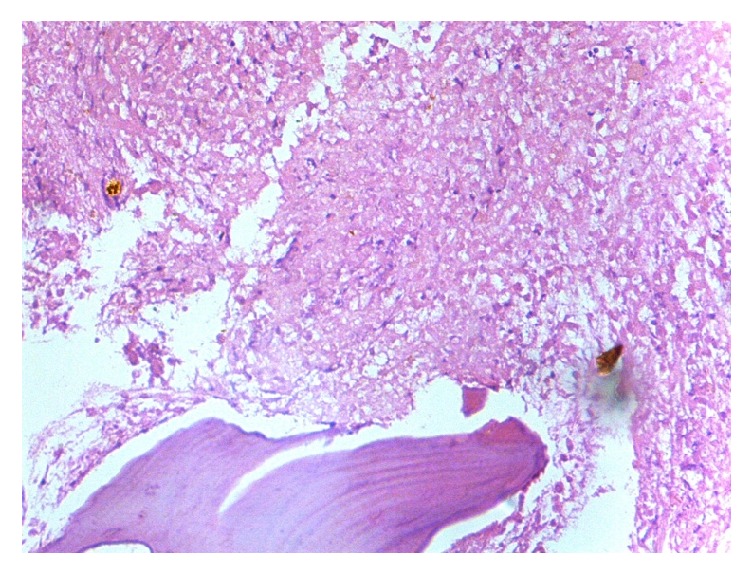
Post therapy histological section of bone marrow biopsy showing extensive necrosis (400x magnification).

Apart from these findings, two patients showed erythroid hyperplasia, 2 patients showed marrow hypoplasia (Figure 2), 1 showed marrow necrosis (Figure 3), and 4 patients showed presence of lymphoid aggregates in bone marrow biopsy.

The most common final MMR score was score 0 (n=17, 58.62%), followed by score 1 (n=8, 27.58%), score 3 (n=4, 13.79%), and score 2 (n=0). Bone marrow morphological response observed is shown in Table 3.

### 4.5. Correlation between MMR Score and Hematological Response

All patients who achieved CHR (n=15) showed a MMR score of 0 (86.67%) or 1 (13.33%). Out of 12 patients having PHR, 10 (83.33%) showed a MMR score of 0 (33.33%) or 1 (50%) and 2 (16.67%) showed a MMR score ≥2. All patients having NHR (n=2) showed a MMR score of ≥ 2. The correlation of morphological response with hematological response is shown in Table 4.

### 4.6. Correlation of MMR Score with Molecular Response

15/22 (68.18%) patients with MMR score 0 or 1 showed complete or early molecular response while 7/22 (31.82%) showed suboptimal molecular response (BCR-ABL transcripts >10%). All the patients with MMR score ≥ 2 (n=4) showed suboptimal molecular response. The correlation of morphological response with molecular response is shown in Table 5.

### 4.7. Assessment of Angiogenesis

**Figure 4:**
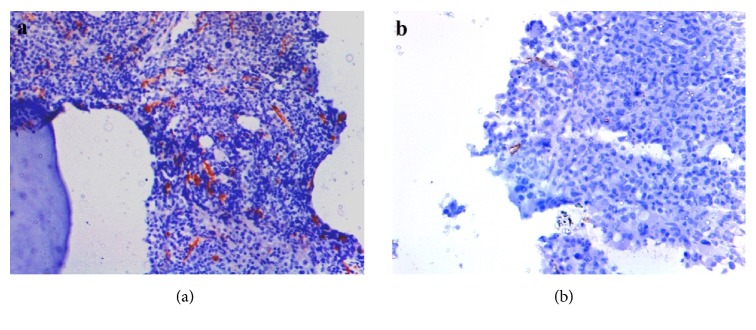
(a) Pre therapy immunohistological section of bone marrow biopsy stained for CD34 (400x magnification) showing microvessels with or without lumen which are stained in brown (mean MVD = 24). (b) Post therapy immunohistological section of bone marrow biopsy of same patient showing reduced number of microvessels (mean MVD = 3.44).

MVD of 30 patients was obtained at the time of diagnosis and the mean MVD was 14.69 ± 5.28. MVD assessment of one patient in blast crisis could not be done at the time of diagnosis as microvessels were obscured by blasts. However, we could assess MVD of the same patient at 3 months of follow-up as blast counts had decreased. Mean MVD of 29 patients could be obtained at 3 months of follow-up after treatment (2 patients died before 3 months of follow-up) and was found to be 4.85±2.80. Initial (14.87 ± 5.36) and final (4.98 ± 2.77) mean MVD of only 28 patients was available. Mean difference in initial and final MVD levels of these 28 patients was found to be 9.89. A drop of 66.51% in posttreatment MVD levels was found at 3 months of follow-up; this change was found to be statistically significant (t=9.483, p<0.001). Mean MVD of 10 control samples was 6.32 ± 1.64, which was significantly lower than mean MVD 30 CML patients (14.69 ± 5.28) at the time of diagnosis (t =4.89, p<0.001).  Figure 4 shows decrease in MVD in a patient on imatinib therapy.

## 5. Discussion

51.7% of patients evaluated in this study achieved CHR after 3 months of imatinib therapy. Narang N et al.** [**10] evaluated sequential hematological parameters at ≤ 1 month, 3 months, 6 months, and 12 months after imatinib therapy and observed that 80% of patients achieved CHR at 3 months. Srinivas BH et al. [10] observed CHR in 15/37 (40.5%) of their patients on first follow-up at 8 months after imatinib therapy.

Significant improvement in bone marrow morphology was seen after imatinib therapy in this study.  Table 6 compares the pre and post therapy morphological findings in this study with similar studies.

In our study, all patients showed some degree of marrow fibrosis at presentation. The pre therapy grade of fibrosis of majority of patients (61.29%) was Grade 2, followed by Grade 3 (25.81%) and Grade 1 (12.9%). The occurrence of myelofibrosis at the time of presentation has been observed in 30-96.15% of CML patients in various studies [11, 12]. This variation may be due to the variability in the definitions of fibrosis, stains used for assessing fibrosis, and time of presentation to the medical facility after the onset of CML [11]. The higher incidence of bone marrow fibrosis at presentation in our study is probably because of a predominantly rural population of patients who often present late in the course of the disease to our referral institution. Most studies in the past have graded myelofibrosis according to the system described by Bauermeister DE (1971), Grades 0-4 [13]. The European consensus on grading bone marrow fibrosis and assessment of cellularity (2005) which has been used in this study, recommended standardization of grading of myelofibrosis for the purposes of accuracy and easy reproducibility [9].

In this study, 72.42% of patients had decrease in grade of fibrosis at 3 months of follow-up and 17.24% did not show any change in grade of fibrosis while 10.34% of patients showed progression of fibrosis grade. Bueso-Ramos CE et al. (2004) evaluated pre and post-imatinib therapy bone marrow fibrosis grades in 40 patients who had treatment failure with interferon-*α* and were then put on imatinib therapy. They observed some degree of myelofibrosis in all patients (100%) prior to the start of imatinib therapy, Grade 1 in 1 patient (2%), Grade 2 in 8 patients (20%), Grade 3 in 16 patients (40%), and Grade 4 in 15 patients (37%). Posttherapy fibrosis grades could be evaluated at follow-up at 3 months in 23 patients, at 6–9 months in 1 patient, at 11–12 months in 8 patients, at 14–18 months in 4 patients, and after 24 months in 4 patients. They observed reduction in grade of fibrosis by at least one grade in 85% of patients [14]. Narang N et al. (2017) evaluated marrow fibrosis in 60 patients prior to imatinib therapy and were able to evaluate fibrosis grades in 48 patients at 6 months of follow-up and 30 patients at 12 months of follow-up. They observed significant improvements in fibrosis grades at 6 months which continued at 12 months of follow-up (repeated ANOVA test, p=0.04) [15]. The above findings suggest that significant decrease in fibrosis grades is evident at 3 months after imatinib therapy and further improvement may occur after longer durations of therapy (12 months) as documented by Narang N et al. (2017). In our study, patients who had progression of fibrosis grade (n=3) had suboptimal molecular response with BCR-ABL transcript levels > 10%.

Several mechanisms have been proposed for myelofibrosis in CML patients. Marrow fibrosis in CML may be mediated by platelet derived growth factor (PDGF) released by megakaryocytes which stimulates fibroblasts [16]. A role of cellular oncogene c-sis translocated from chromosome 22 to chromosome 9 whose protein is like PDGF has been suggested [14]. Imatinib is a tyrosine kinase inhibitor that inhibits the BCR-ABL1 tyrosine kinase, the constitutive abnormal tyrosine kinase created by 9:22 translocation [17]. In addition to its activity against BCR-ABL tyrosine kinase, it also inhibits PDGFR and therefore may have a role in suppression of marrow fibrosis [18–20].

We observed erythroid hyperplasia in 2/29 (6.89%) of our patients after imatinib therapy. 2 (6.89%) patients showed marrow hypoplasia, 1 (3.45%) showed marrow necrosis, and 4 (13.79%) patients showed presence of lymphoid aggregates in bone marrow biopsy. Erythroid hyperplasia after imatinib therapy has also been observed in other studies with an incidence range of 13.51%-36.7% [10, 21]. Marrow hypoplasia has been reported with an incidence of 10%-54.4% after imatinib therapy [15, 21, 22]. Marrow necrosis is an extremely rare event after imatinib therapy and it may occur due to the apoptotic effect of imatinib therapy and release of prothrombotic cellular material [23, 24]. Lymphoid aggregates were observed in 42% cases after imatinib therapy in another study [25].

In this study, we observed a statistically significant correlation between MMR score and hematological response (Table 4) and between MMR score and molecular response (Table 5). It was observed that patients with MMR score ≥ 2 after imatinib therapy are more likely to have suboptimal molecular response with BCR-ABL transcripts >10%.

The mean MVD of 10 control samples in this study was 6.32 ± 1.64, which was significantly lower (p<0.001) than mean MVD of 30 CML patients (14.69 ± 5.28) at the time of diagnosis. This suggests that the MVD may have an important role in the formation of tumor microenvironment and pathogenesis of tumor. We observed a statistically significant (t=9.483, p<0.001) drop of 66.51% in post treatment mean bone marrow MVD levels in CML patients after 3 months of imatinib therapy. This suggests an antiangiogenic role of imatinib. Lokeshwar NM et al. [26] also observed significant reduction in MVD in CML patients after imatinib therapy (Table 7). Kvasnicka HM et al. observed that decrease in microvascularization after imatinib therapy is associated with the reversal of myelofibrosis [27].

In CML patients, increased angiogenesis is the result of BCR-ABL oncogene induced increased expression of VEGF gene [28]. Reduction in plasma VEGF concentration has been observed after 6 months of imatinib therapy in CML patients [29]. The mechanisms by which imatinib decreases angiogenesis in CML are not well known. Decrease in angiogenesis and plasma VEGF concentration after imatinib therapy may partly be due to the anti BCR-ABL effect of imatinib [30]. Other mechanisms have also been proposed. Raimondi C et al. in* in vivo* studies in mice retina demonstrated that neuropilin 1 (NRP1) which is a receptor on endothelial cells for VEGF regulates angiogenesis in a VEGF independent fashion via ABL1 tyrosine kinase [31].

## 6. Conclusion

Apart from hyperproliferation of myeloid precursors, the bone marrow microenvironment in CML is also altered. There is significant increase in marrow fibrosis and neovascularization. Imatinib mesylate therapy not only decreases the marrow cellularity, but also helps towards normalization of the bone marrow microenvironment by reducing fibrosis and angiogenesis.

## Figures and Tables

**Table 1:** Criteria for assessment of morphological response.

**Parameter**	**Finding**	**Points**
**Cellularity**	not increased for age	0
	increased for age	1

**M:E ratio**	≤ 4:1	0
	> 4:1	1

**Fibrosis**	grades 0 to 2	0
	grade 3	1

**Abnormal megakaryocytes**	< 10% of all megakaryocytes	0
	≥ 10% of all megakaryocytes	1

**Type of tap**	not dry	0
	dry	1

**Percentage of blasts**	dry tap	0
	not dry and < 5%	0
	not dry and ≥ 5%	1

**Percentage of basophils**	dry tap	0
	not dry and < 1%	0

M:E ratio: myeloid: erythroid ratio.

**Table 2:** Hematological response.

**Phase at Diagnosis**	**No. of patients**	**Follow-up: No. of patients (**%**)**		
		**CHR**	**PHR**	**NHR**
**Chronic phase**	25	13 (52%)	10 (40%)	2 (8%)

**Accelerated phase **	2	1 (50%)	1 (50%)	

**Blast Phase**	4 (2 died)	1 (50%)	1 (50%)	

CHR: complete hematological response, PHR: partial hematological response, and NHR: nil hematological response.

**Table 3:** Bone marrow morphology findings at the time of diagnosis and at 3 months after treatment follow-up.

**Morphological parameter**		**At the time of diagnosis**	**At 3 months of follow-up**
		**(N=31)**	**(N=29)**
**Increased cellularity**		31 (100%)	4 (13.79%)

**M:E ratio > 4:1**		31 (100%)	4 (13.79%)

**Abnormal megakaryocytes**		31 (100%)	5 (17.24%)

**Fibrosis**	**Grade 0**	0	6 (20.69%)
	**Grade 1**	4 (12.9%)	12 (41.38%)
	**Grade 2**	19 (61.29%)	9 (31.03%)
	**Grade 3**	8 (25.80%)	2 (6.89%)

**Blast count > 5**%		10 (32.25%)	1 (3.44%)

**Basophils > 1**%		22 (70.96%)	5 (17.24%)

M:E ratio: myeloid: erythroid ratio

**Table 4:** Correlation of marrow morphological response (MMR) score with hematological response.

**MMR Score**	**No. of patients**	**CHR**	**PHR**	**NHR**
	**(N=29)**	**(n=15)**	**(n=12)**	**(n=2)**
**0**	17 (58.62%)	13 (86.67%)	4 (33.33%)	0

**1**	8 (27.59%)	2 (13.33%)	6 (50%)	0

**2**	0	0	0	0

**3**	4 (13.79%)	0	2 (16.67%)	2 (100%)

MMR: marrow morphological response, CHR: complete hematological response, PHR: partial hematological response, and NHR: nil hematological response. Fischer exact test was used for statistical association, p<0.001.

**Table 5:** Correlation of marrow morphological response (MMR) score with molecular response.

**MMR score**	**No. of patients (N=26)**	**Complete or early molecular response**	**BCR-ABL transcripts >10**%
**0**	15 (57.70%)	11 (73.33%)	4 (26.67%)

**1**	7 (26.92%)	4 (57.14%)	3 (42.86%)

**2**	0	0	

**3**	4 (15.38%)	0	4 (100%)

Fischer exact test was used for statistical association, p<0.05.

**Table 6:** Comparison of bone marrow morphological changes between the present study and previous studies.

**Morphological parameters**	**BH Srinivas et al.**		**Present study**	
	**(2012)**		**(2017)**	
	**Initial**	**8 months of follow-up**	**Initial**	**Follow-up**

**Increased cellularity**	100%	20.4%	100%	13.8%

**M:E ratio > 4:1**	100%	35.1%	100%	27.6%

**Blast > 5**%	17%	10.6%	27.6%	3.4%

**Basophil >1**%	61%	25.5%	93.1%	41.3%

**Abnormal megakaryocytes**	100%	27.5%	100%	24.1%

**MMR score**				
**≤ 1**		66.34%		86.20%
**≥2**		33.66%		13.79%

M:E ratio: myeloid: erythroid ratio and MMR: marrow morphological response.

**Table 7:** Comparison of mean MVD in bone marrow before and after imatinib therapy between various studies.

**STUDY**	**Control mean MVD**	**Initial mean MVD**	**Follow-up mean MVD**	
**Lokeshwar NM et al. (2006) **	3.6	8.25	4 (6 months)	p = 0.04

**Present study (2017)**	6.32	14.87	4.98 (3 months)	p < 0.001

MVD: microvessel density.

## Data Availability

The data used to support the findings of this study are available from the corresponding author upon request.
